# Mycotoxin Co-Occurrence in Michigan Harvested Maize Grain

**DOI:** 10.3390/toxins14070431

**Published:** 2022-06-24

**Authors:** Katlin Fusilier, Martin I. Chilvers, Victor Limay-Rios, Maninder P. Singh

**Affiliations:** 1 Department of Plant, Soil and Microbial Sciences, Michigan State University, East Lansing, MI 48824, USA; blaineka@msu.edu (K.F.); chilvers@msu.edu (M.I.C.); 2 Ridgetown Campus, University of Guelph, Ridgetown, ON NOP 2CO, Canada; vlimayri@uoguelph.ca

**Keywords:** mycotoxin, deoxynivalenol, co-occurrence, maize

## Abstract

Mycotoxins are secondary metabolites produced by fungi that, depending on the type and exposure levels, can be a threat to human and animal health. When multiple mycotoxins occur together, their risk effects on human and animal health can be additive or synergistic. Little information is known about the specific types of mycotoxins or their co-occurrence in the state of Michigan and the Great Lakes region of the United States. To understand the types, incidences, severities, and frequency of co-occurrence of mycotoxins in maize grain (*Zea mays* L.), samples were collected from across Michigan over two years and analyzed for 20 different mycotoxins. Every sample was contaminated with at least four and six mycotoxins in 2017 and 2018, respectively. Incidence and severity of each mycotoxin varied by year and across locations. Correlations were found between mycotoxins, particularly mycotoxins produced by *Fusarium* spp. Environmental differences at each location played a role in which mycotoxins were present and at what levels. Overall, data from this study demonstrated that mycotoxin co-occurrence occurs at high levels in Michigan, especially with mycotoxins produced by *Fusarium* spp., such as deoxynivalenol.

## 1. Introduction

Mycotoxins are secondary metabolites produced by filamentous fungi, particularly in the genera *Alternaria*, *Aspergillus*, *Fusarium*, or *Penicillium* [1,2]. These toxins can be harmful to both humans and animals. Acute, short-term exposure can lead to varied effects depending on the type of mycotoxin present [3]. Although acute exposure is important, grain with high levels of mycotoxin usually does not enter the channel of trade. More often, undetected chronic exposure to low doses of mycotoxins is found. This chronic exposure can lead to reduced weight gain, diminished productivity, and increased susceptibility to infections in animals [3].

In maize grain (*Zea mays* L.), mycotoxin contamination in the field occurs through fungal ear rot infections. In many cases, ear infections occur when fungal spores land on maize silks and grow down into the ear or when spores enter through damage from insects or birds. Because mycotoxin-producing fungi may produce more than one type of mycotoxin, and grain can become infected with multiple fungal species at a time, it is important to understand the frequency of mycotoxin co-occurrence [1]. When mycotoxins co-occur, they can interact and have antagonistic, additive, less than additive, or synergistic effects [1]. However, information on incidence, severity, and effects of contamination with co-occurring mycotoxin is lacking in maize grain, especially in the Great Lakes Region of the United States (U.S.).

Although there are thousands of mycotoxins currently identified, only a few are regulated for food and animal feed across the world. These regulated mycotoxins are aflatoxins, ochratoxin A, zearalenone (ZEN), fumonisins, and select trichothecenes, including deoxynivalenol (DON), HT-2, and T-2 toxins [4,5]. Although these mycotoxins are considered the most important for human and animal health and safety, masked and other emerging mycotoxin are also becoming important. Masked mycotoxins such as deoxynivalnol-3-β-glucoside (D3G) are biologically modified metabolites conjugated by plant defense mechanisms [6]. Emerging mycotoxins are a group of mycotoxins with no current regulations that are very chemically diverse from one another [4]. Masked and emerging mycotoxins are important, as they may interact with regulated mycotoxins present in maize grain.

To reduce the incidence and occurrence of mycotoxins entering grain markets, the U.S. and other countries set limits on mycotoxins in grain sold in the marketplace. The U.S. Food and Drug Administration (FDA) has set action levels for aflatoxins and advisory levels for DON and fumonisins [7,8,9]. As a result, these mycotoxins are regularly assessed in food and feed. Outside of the U.S., all countries with mycotoxin regulations have limits for aflatoxin B1 or the total aflatoxin level in food and/or feed [10]. Examples of other mycotoxins regulated in various countries include aflatoxin M1, diacetoxyscirpenol (DIAS), T-2 toxin (T-2), HT-2 toxin (HT-2), and ZEN (Table 1) [10]. Limited information is available on their frequency in the U.S. maize supply because their occurrence is not monitored.

Because many fungal infections occur during the time of silking, when fungal spores land on the silks, germinate, and propagate into the developing ear [11], environmental conditions around the time of silking and ear development are important in determining mycotoxin accumulation and co-occurrence in harvested grain. Fungal infections can also occur though other modes of entry, such as though wounds caused by ear-feeding insects such as western bean cutworm (*Striacosta albicosta*) [12], potentially leading to an increase in mycotoxin concentrations in maize grain [13,14,15].

**Table 1:** Complete list of mycotoxins tested along with abbreviations, fungal species that produce specific mycotoxin, and regulatory limits worldwide for each mycotoxin across crops.

Mycotoxin	Abbreviation	Produced by:	Regulations
Aflatoxin	AB1	*Aspergillus flavus*, *A. parasiticus*, and *A. nominus* [16]	Food
			Aflatoxin B1: 61 countries; 1 μg kg^−1^ to 20 μg kg^−1^ [17]
			Total Aflatoxins: 76 countries; 0 μg kg^−1^ to 35 μg kg^−1^ [17]
			Feed
			Aflatoxin B1: 39 countries; 5 μg kg^−1^ to 50 μg kg^−1^ [17]
			Total Aflatoxins: 21 countries; 5 μg kg^−1^ to 50 μg kg^−1^ [17]
Beauvericin	BEA	*Beauveria bassiana* and *Fusarium* spp. [18,19,20]	
Diacetoxyscirpenol	DIAS	*Fusarium* spp. esp. *F. poae*, *F. equiseti*, *F. sambucinum*, and *F. sporotrichioides* [20,21]	
Deoxynivalenol	DON	*Fusarium graminearum* and *F.culmorum* [20]	37 countries; 300 μg kg^−1^ to 2000 μg kg^−1^ [17]
deoxynivalenol 3-β-D -glucoside	D3G	*Fusarium* spp. [22]	
15-acetyl-deoxynivalenol	15-ADON	*Fusarium graminearum* and *F. culmorum* [20]	
3-acetyl-deoxynivalenol	3-ADON	*Fusarium graminearum* and *F. culmorum* [20]	
Enniatin A	ENNA	*Fusarium* spp. [18]	
Enniatin A1	ENNA1	*Fusarium* spp. [18]	
Enniatin B	ENNB	*Fusarium* spp. [18]	
Enniatin B1	ENNB1	*Fusarium* spp. [18]	
Fumonisin B1	FB1	*Fusarium* spp. [20,23]	Total Fumonisins: 6 countries; 1000 μg kg^−1^ to 3000 μg kg^−1^ [17]
Fumonisin B2	FB2	*Fusarium* spp. [20,23]	
Fumonisin B3	FB3	*Fusarium* spp. [20,23]	
Fusarenon-X	FUSX	*Fusarium* spp. [24]	
HT-2 Toxin	HT-2	*Fusarium* spp. esp. *F. sporotrichioides*, *F. acuminatum*, and *F. poae* [20]	T-2 + HT-2: EU; 15 μg kg^−1^ to 2000 μg kg^−1^ [25]
Moniliformin	MON	*Fusarium* spp. esp. *F. subglutinans*, *F. groliferatum*, *F. avenaceum*, and *F. tricinctum* [18,20]	
Nivalenol	NIV	*Fusarium cerealis*, *F. poae*, *F. graminearum*, and *F. culmorum* [20,24]	
T-2 Toxin	T-2	*Fusarium* spp. esp. *F. sporotrichioides*, *F. acuminatum*, and *F. poae* [20]	T-2 + HT-2: China, Iran, Canada, EU; 15 μg kg^−1^ to 2000 μg kg^−1^ [25]
Zearalenone	ZEN	*Fusarium* spp. esp. *F. graminearum*, *F. culmorum*, *F. cerealis*, *F. equiseti*, *F. crookwellense*, and *F. semitectum* [20,26]	16 countries; 50 μg kg^−1^ to 1000 μg kg^−1^ [17]

Due to limited information available on multiple mycotoxins in maize grain in Michigan and across the Great Lakes Region, the objectives of this study were to determine the type of mycotoxins present in Michigan maize, their level, and frequency of occurrence and co-occurrence with one another in relation to environmental variability.

## 2. Results

Mycotoxins tested included aflatoxin, beauvericin (BEA), DIAS, DON, D3G, 15-acetyl-deoxynivalenol (15-ADON), 3-acetyl-deoxynivalenol (3-ADON), enniatin A (ENNA), enniatinA1 (ENNA1), enniatin B (ENNB), enniatin B1 (ENNB1), fumonisin B1 (FB1), fumonisin B2 (FB2), fumonisin B3 (FB3), fusarenon-X, HT-2, moniliformin (MON), nivalenol, T-2, and ZEN. Out of the mycotoxins tested, four toxins were not found in either year: aflatoxin, diacetoxyscirpenol, fusarenon-X, and nivalenol.

### 2.1. Frequency of Mycotoxin Co-Occurrence

**Figure 1:**
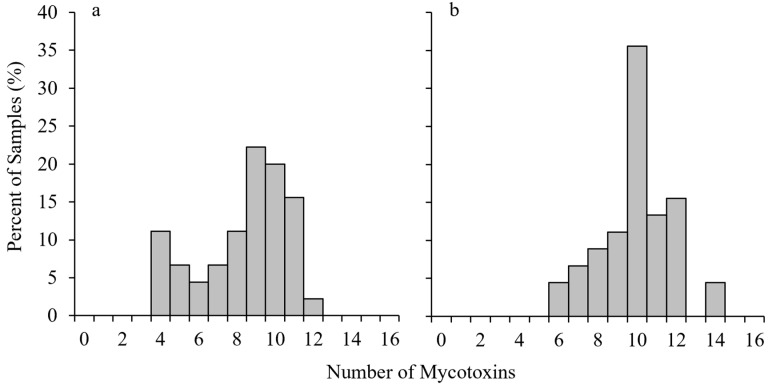
Distribution of multiple mycotoxin occurrence in 45 samples in each of nine locations throughout Michigan during 2017 (**a**) and 2018 (**b**) growing seasons. Bars indicate the percentage of samples with a certain number of mycotoxins present. Samples were tested for 20 different mycotoxins.

Co-contamination by multiple mycotoxins was found to be highly prevalent in Michigan maize grain. Contamination with more than one mycotoxin was observed in all samples tested. Out of the 20 mycotoxins tested, each sample in the study was contaminated with at least four different mycotoxins in 2017 and six different mycotoxins in 2018. The maximum number of contaminates per sample was 12 in 2017 and 14 in 2018. The average number of individual mycotoxin contaminates per sample was 8.4 in 2017 and 10.0 in 2018 (Figure 1).

### 2.2. Mycotoxin Incidence and Severity

Results from this study indicate that the overall incidence of mycotoxin contamination in Michigan is relatively high (Table 2 and Table 3). Several mycotoxins had particularly high incidences, showing up in a large number of samples tested. In 2017, DON, ENNA, ENNB, FB1, and FB2 were found in more than 80% of samples tested, whereas BEA, DON, D3G, 15-ADON, FB1, FB2, FB3, and ZEN were found in greater than 80% of samples in 2018.

#### 2.2.1. Mycotoxin Correlations

**Figure 2:**
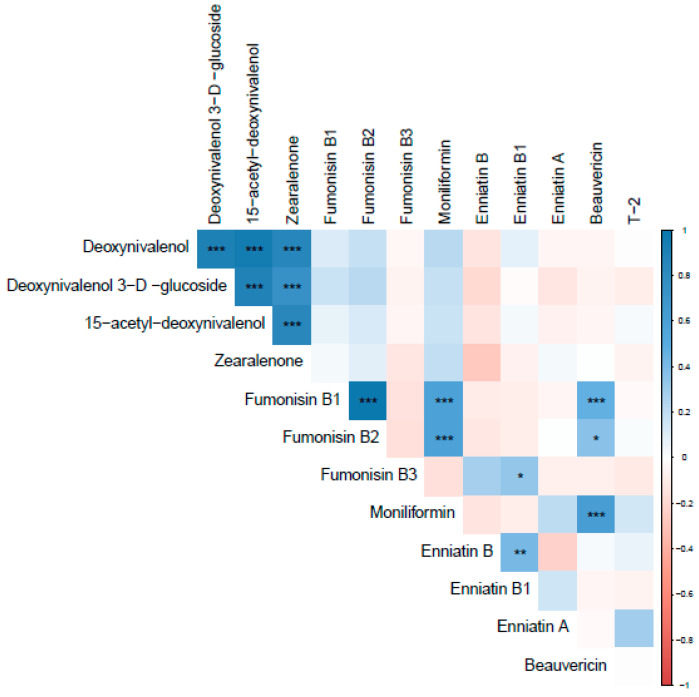
Pearson’s correlation coefficient for mycotoxins in 2017 samples. ***, **, and * represent *p*-values of 0.001, 0.01, and 0.05, respectively. Blue colors indicate positive correlations, while red indicates negative correlations.

Various mycotoxins were found to be correlated in both 2017 and 2018. In 2017, DON was strongly correlated with D3G, 15-ADON, and ZEN (r = 0.90, 0.95, and 0.86, respectively). Fumonisin B1 and FB2 were also found to be strongly correlated (r = 0.99). A moderate correlation was found between MON and FB1, FB2, and BEA (r = 0.60, 0.59, and 0.62, respectively) (Figure 2).

**Figure 3:**
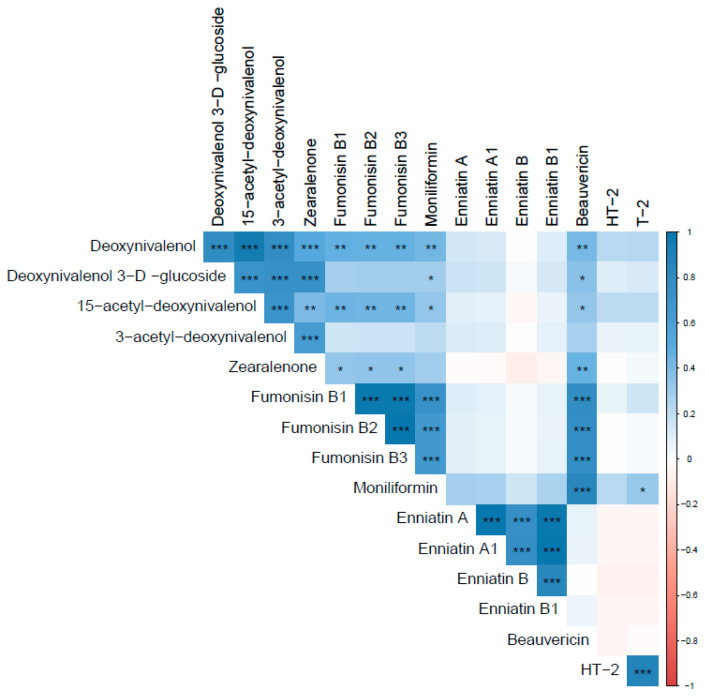
Pearson’s correlation coefficient for mycotoxins in 2018 samples. ***, **, and * represent *p*-values of 0.001, 0.01, and 0.05, respectively. Blue colors indicate positive correlations, while red indicates negative correlations.

In 2018, more correlations were found between mycotoxins (Figure 3). Strong correlations were found between DON and D3G (r = 0.80) and DON and 15-ADON (0.96). A moderately strong correlation was found between DON and 3-ADON (0.77). Moderate correlations were found between DON and ZEN, FB1, FB2, FB3, MON, and BEA (r = 0.53, 0.47, 0.47, 0.46, 0.43, respectively). Strong correlations were found between FB1 and FB2 (r = 0.99) and FB1 and FB3 (r = 0.99). All three fumonisins (FB1, FB2, and FB3) had strong correlations with MON (r = 0.73, 0.67, and 0.67, respectively). Beauvericin was also found to have a strong correlation with FB1, FB2, FB3, and MON (r = 0.78, 0.77, 0.77, and 0.85, respectively). Enniatin B1 was found to be strongly correlated with ENNB, ENNA, and ENNA1 (r = 0.84, 0.99, and 0.99, respectively). Further, in 2018, a strong correlation was found between HT-2 and T-2 (r = 0.87).

#### 2.2.2. Specific Mycotoxins

Many of the mycotoxins commonly found in the study were produced by *F. graminearum.* Deoxynivalenol was the most commonly occurring mycotoxin in the study, with 93% of samples contaminated in 2017 (Table 2) and 100% contaminated in 2018 (Table 3). Additionally, DON was found at a high quantity in many of the samples, and average DON values in both 2017 and 2018 were greater than 1000 µg kg^−1^. Deoxynivalenol was found over 1000 µg kg^−1^ in 42% of samples in 2017 and 80% of samples in 2018. It was also found to be over 5000 µg kg^−1^ in 2% of samples in 2017 but 38% of samples in 2018. Deoxynivalenol 3-β-D-glycoside is considered a masked mycotoxin and was present in a high number of samples, with 73% and 100% of samples contaminated in 2017 (Table 2) and 2018, respectively (Table 3). The mycotoxins 15-ADON and 3-ADON are derivatives of DON [20]. The mycotoxin 15-ADON was found in both 2017 and 2018, while 3-ADON was only found in 2018. Zearalenone, also produced by *F. graminearum*, was found in 69% of samples in 2017 and 100% of samples in 2018. At least 16 countries have ZEN regulations in place with limits for ZEN in maize and other cereals ranging from 50 to 1000 µg kg^−1^ [26]. In 2017, 36% of samples were over 50 µg kg^−1^, and 7% were over 1000 µg kg^−1^. During the 2018 growing season, 64% were over 50 µg kg^−1^, while 16% were over 1000 µg kg^−1^.

The fumonisins, namely FB1, FB2, and FB,3 are regulated in the U.S. as the total concentration of all three analogs [9]. One sample in the study was over the FDA limit of 20,000 µg kg^−1^ for total fumonisin concentration for swine [9]. This sample was from Huron in 2018 and had a total fumonisin concentration of 82,236 µg kg^−1^. The fumonisins FB1, FB2, and FB3 are often found occurring together [27]. Contamination of samples by all three analogs, FB1, FB2, and FB3, was found in both years. All three analogs were found in 56 and 89% of samples in 2017 and 2018, respectively. Moniliformin was found in both years of the study though with higher incidences and concentrations in 2018 compared to 2019.

T-2 and HT-2 toxins are not regulated in the U.S. In 2017, only T-2 was found in samples, while in 2018, both T-2 and HT-2 were present. Certain countries regulate T-2 toxin individually, while others regulate the total concentration of T-2 and HT-2. The European Union’s lowest limit for T-2 and HT-2 is 15 µg kg^−1^ [25]. In both 2017 and 2018, only 9% of samples were found to be above 15 µg kg^−1^.

The enniatins and BEA are structurally related mycotoxins [28]. BEA was found most often across the two years with 78% contamination in 2017 (Table 2) and 100% contamination in 2018 (Table 3). In 2017, higher levels of enniatins were found compared to 2018. Across both years ENNA and ENNB were found at higher levels than ENNA1 and ENNB1. In 2017, total enniatin concentration of ENNA, ENNA1, ENNB, and ENNB1 ranged between 0.1 and 1.36 µg kg^−1^, with 100% of the samples having enniatins present. In 2018, total enniatin concentration ranged between less than the LOD to 59.40 µg kg^−1^, with 27% of the samples having enniatins present.

### 2.3. Variability across Locations

In 2017, DON, D3G, ZEN, and ENNA levels varied significantly by location (Table 4). Washtenaw had the highest DON levels but was not significantly different from Cass, Allegan, Branch, or Montcalm. Likewise, D3G followed a similar pattern as DON, with Washtenaw having the highest levels of D3G but not different from Allegan, Cass, and Branch. The mycotoxin ENNA was found at all nine sites, with Saginaw, Washtenaw, and Ingham having the highest levels of ENNA. Zearalenone was found to be the highest in Washtenaw, while only very low levels were found in Huron, Ingham, Mason, Montcalm, and Saginaw.

In 2018, DON, D3G, HT-2, and T-2 varied significantly by location (Table 5). Huron had the highest DON levels but was not significantly different from Ingham, Mason, Montcalm, or Saginaw. In addition, D3G levels were found to be the highest in Saginaw and lowest in Branch and Washtenaw. Significantly higher levels of T-2 were found in Huron than all other locations. HT-2 only occurred in two out of nine locations in 2018.

### 2.4. Environmental Conditions

In 2017, average temperatures in July varied across the state, with some locations experiencing higher than average temperatures, while others were lower than average (Table 6). During the last half of August and early September, temperatures across the state were generally cooler than normal. This resulted in lower than normal temperature at all locations in August. Average September temperatures were higher than normal at all locations except Branch. This increase in temperature in September can mostly be attributed to higher than normal temperatures across the state during the last part of the month. Rainfall was greater than normal in Mason in July 2017. Besides this exception, rainfall was lower than normal at all other locations between July and September. Though many of the months were lower than normal, there was still great variability in rainfall between locations. July through September precipitation totals ranged between 75.6 mm and 200 mm (Table 6).

Environmental conditions also varied between locations in 2018. At the majority of locations, temperatures trended higher in 2018 throughout most of July and August compared to 2017. These July and August temperatures were greater than the 30-year average at all locations except Montcalm. Average temperatures in September were higher than the 30-year average at all locations. Temperatures in September trended lower at the end of the month when compared to 2017. Precipitation levels were generally higher in 2018 compared to 2017. July through September precipitation totals ranged between 158 mm and 326 mm in 2018. Rainfall was lower than normal at all locations in July and higher than normal at all locations in August. In September, precipitation was higher than normal in Ingham, Mason, and Montcalm, while all other locations had lower rainfall than normal. Overall, July through September precipitation was higher than normal in Mason, Montcalm, and Saginaw and lower than normal at all other locations (Table 6).

### 2.5. Western Bean Cutworm Incidence

Western bean cutworm incidence was calculated for each of the locations in 2017 and 2018. Western bean cutworm incidence in 2017 was significantly higher in Washtenaw compared to all other locations. In 2018, there were no locations that were statistically different from one another (Table 7).

## 3. Discussion

Many different types of mycotoxins were found across the state of Michigan in this study. Not only was the incidence of these mycotoxins high, but the concentration of mycotoxin contamination was also high in certain scenarios. Moreover, the levels of mycotoxin co-occurrence were found to be high across the two years of study.

High levels of multiple mycotoxin contamination were shown to occur in this study, with all samples testing positive for at least four different mycotoxins. Results from a worldwide mycotoxin survey between 2009 and 2011 found that North, Central, and South America had 40% of finished feed samples testing positive for contamination from multiple mycotoxins using ELISA or HPLC testing for aflatoxins, ZEN, DON, fumonisins, and ochratoxins [29]. Studies from maize in Tanzania reported that 87% of samples were contaminated with more than one mycotoxin when tested using HPLC/TOFMS [30]. Based on this information, multiple mycotoxin co-occurrence seems to be common in maize and other feedstuffs across many geographic areas, including the U.S. Great Lakes Region.

Both the incidence and severity of mycotoxins influence the levels of contamination. The mycotoxins DON, BEA, FB1, D3G, ZEN, FB2, and FB3 were all found in greater than 80% of the samples surveyed across the two years of the study. This is similar to findings from a neighboring region (Ontario, Canada), Belgium, as well as a seven-year mycotoxin survey in the U.S. where mycotoxins with the highest incidence were DON, FB1, and ZEN [31,32,33]. Mycotoxins found in these studies are produced mostly by *Fusarium* spp. [20,23,26], indicating that mycotoxin production by *Fusarium* spp. in maize grain may be a common issue in the temperate zone. In this study, aflatoxin was not found in any sample, indicating that aflatoxins are not currently an issue in the Great Lakes Region. Aflatoxins were also found to be absent in a farm survey conducted in Northern Europe [34]. Rate of mycotoxin production can also vary with the fungal genera and within strains of a fungal species [3].

Correlations between mycotoxins was also found in both years of the study. Many of the mycotoxins found to be correlated with one another are produced by *Fusarium* spp., including DON, its derivatives, ZEN, the fumonisins, MON, and BEA. Correlations between the three fumonisin analogs were also found in the study. These data indicated that mycotoxins produced by a fungal species or different species of the same genus (Fusarium) are often found occurring together. Similar correlations were observed in mycotoxin surveys carried out in the U.S. and Italy [33,35]. These correlations may help in determining when mycotoxins could co-occur.

Understanding the role of environmental factors on ear rot occurrence and associated mycotoxin accumulation is important in efforts to reduce the co-contamination of mycotoxins [36]. Infection through maize silks is an important point of entry for many ear rot causing fungal pathogens [12]. Environmental conditions before, during, and after the silking period are important for fungal infection [12]. Differences in environmental conditions around silking between years and locations may factor into which mycotoxins are present and at what concentrations. At the study locations, 2018 trended hotter throughout the majority of the seasons and locations. In addition, precipitation was higher around silking in August 2018. Between locations, weather patterns were variable around silking times, which may have caused mycotoxins to vary by location as various fungal species prefer specific environmental conditions. Knowing the weather patterns around and following silking can help growers in predicting which mycotoxins may be present in their fields and at what concentrations as each fungal species may favor different weather conditions. Further research is needed in developing predictive models that can assess risk and estimate level of mycotoxin co-occurrence in maize grain based on regional environmental conditions.

In addition, western bean cutworm damage to ears allows for the entry of the fungal pathogen [12]. In 2017, Washtenaw had significantly higher western bean cutworm damage than all other locations. Washtenaw also had the highest concentrations of DON, D3G, ZEN, and ENNA though not significantly different from some other locations. These data showed that the increase in western bean cutworm incidence may lead to an increase in fungal infection, resulting in increased DON and other mycotoxin contamination of maize grain.

Overall, this study has determined that multiple mycotoxin contamination is present at high levels in Michigan and likely throughout the Great Lakes Region just as it has been shown to be present in other areas of the world. Toxins not regularly tested for in the U.S. are present in maize grain, which may be affected by environmental conditions. A broader survey should be conducted to increase knowledge of the occurrence of multiple mycotoxin contamination in maize grain throughout the varied growing environments found in the U.S. corn belt. Moreover, these findings emphasize the importance of research into the effects of mycotoxin co-occurrence on human and animal health. Though concentrations of some detected mycotoxins were low, these should be taken into consideration due to the possible synergistic or additive effects with other mycotoxins occurring in maize grain.

## 4. Materials and Methods

**Figure 4:**
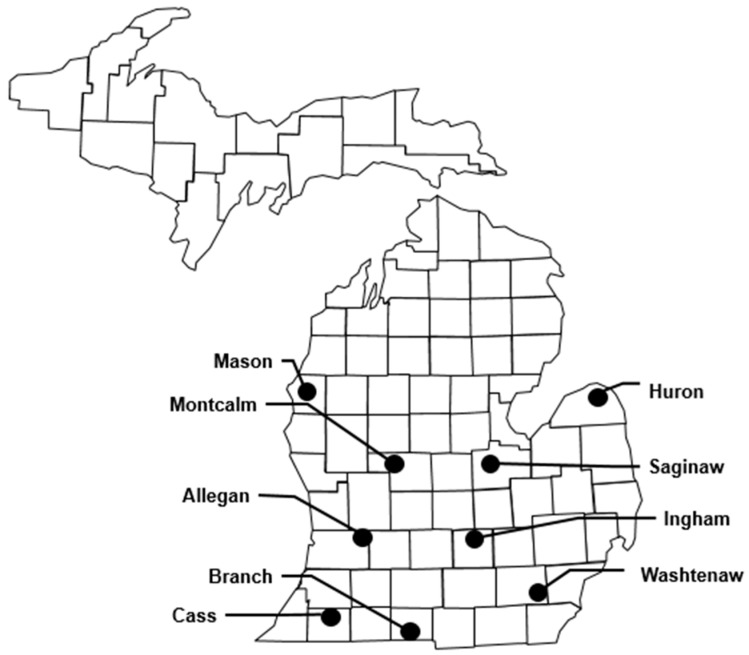
Sampling locations in Michigan during the 2017 and 2018 growing seasons. Maize grain samples were obtained from the same hybrid (2600 growing degree units to black layer) at each location in both years of the study.

Samples were obtained from a larger experiment at nine locations in Michigan in each of the 2017 and 2018 growing seasons [37] for a total of 18 unique environments (Figure 4). Locations were selected to represent the variability of environmental conditions across Michigan. Locations generally had loam or sandy loam soils except Mason 2017 and Washtenaw 2018, which had loamy sand complexes. Branch, Cass, and Mason were under irrigation, while all others were non-irrigated. Most plots were in fields following soybean (*Glycine max* (L.) Merr.) except Huron 2017 and 2018, Branch 2018, Cass 2018, Montcalm 2018, and Washtenaw 2018, which all followed maize. Mason 2017 followed carrots with a rye cover. All plots were planted on commercial farms, and all fungal infection was from natural sources. Plots were planted with a four-row Almaco packet planter (Almaco, Nevada, IA, USA) with row spacing of 0.76 m. Plots measured 6.71 m by 3.05 m with 0.91 m alleyways between plots. Plots were planted between 10 May and 29 May in 2017 and between 9 May and 13 June in 2018. The center two rows of each plot were used for data collection, with the outer two rows acting as a buffer. Average population at locations ranged between 77,219 and 85,586 plants per hectare. Plots were managed according to grower standards for the area. One location (Cass 2018) received a blanket application of Delaro (prothioconazole and trifloxystrobin, Bayer^®^, Research Triangle Park, NC, USA) fungicide via center-pivot irrigation system at 365.56 mL ha^−1^. Delaro is not labeled for the control of ear rots or mycotoxins in maize grain. No other locations in the study were treated with a fungicide.

At each location, samples were collected from one hybrid typical to Michigan growers. The hybrid selected was rated 2600 growing degree days to black layer. This hybrid was chosen because it had “average” tolerance to Gibberella ear rot, Aspergillus ear rot, and Fusarium ear rot as rated by the seed company. The Bt trait package included Lepidoptera proteins Cry1A.105, Cry1Ab2, and Cry1F, which provide protection against several ear-feeding Lepidoptera insects. This includes black cutworm (*Agrotis ipsilon*), corn earworm (*Helicoverpa zea*), European corn borer (*Ostrinia nubilalis*), fall armyworm (*Spodoptera frugiperda*), and stalk borer (*Papaipema nebris*) [38]. Western bean cutworm, the most important ear-feeding insect in Michigan was not controlled by the Bt protein in the hybrid used.

Sample collection at each location occurred in five replicated plots, resulting in 90 samples across the nine locations and two years of study period. For each plot, ten consecutive ears from each of the middle two rows were hand-harvested for a total of 20 ears per plot once kernels reached physiological maturity [39]. Husks were removed, and ears were rated for western bean cutworm incidence, calculated as the percentage of the 20 ears that were damaged by the insect. Ears were then shelled using a Haban Husker-Sheller (Haban Manufacturing, Co., Racine, WI, USA). Shelled kernels from the 20 ears were combined and mixed thoroughly, and a 500 g representative sample was then taken. This sample was ground using a cyclone sample mill (UDY Corporation, Fort Collins, CO, USA) with a 1 mm screen, and a 50 g subsample was submitted for mycotoxin analysis.

Samples were tested for 20 different mycotoxins (Table 1). All mycotoxin detection and quantification, except FB3 and Aflatoxin B1, was performed using an Ionics EP 10 + modified API 365 triple quadrupole mass spectrometer (LC-MS/MS; AB SCIEX, Concord, ON, CA.) system equipped with an electrospray ionization source, in positive and negative polarity, at the University of Guelph, Ridgetown Campus, Ontario, Canada following the method described in [40]. The optimized LC-ESI–MS/MS parameters for FB3 (retention time: 13.02 min) were 706.6 mass-to-charge ratio (*m*/*z*) for precursor ion (declustering potential: 130 V) and 336.3 *m*/*z* (quantifier ion; collision energy: 56 V; cell exit potential: 30 V) and 318.5 *m*/*z* (qualifier ion; collision energy: 47 V; cell exit potential: 45 V) for product ions. For aflatoxin B1 (retention time: 9.0 min), parameters were 312.9 mass-to-charge ratio (*m*/*z*) for precursor ion (declustering potential: 127 V) and 284.9 *m*/*z* (quantifier ion; collision energy: 32 V; cell exit potential: 17 V) and 241.1 *m*/*z* (qualifier ion; collision energy: 50 V; cell exit potential: 17 V) for product ions. Recovery test was performed in triplicate, as previously described, by spiking 5 g of blank ground maize samples at two concentrations (50.0 and 100.0 ng/g). Mean recovery value for FB3 and aflatoxin B1 was 97.7% ± 4.5% (s.d.) and 98.1 ± 6.5% with a LOD of 5.8 and 10.2 n/g, respectively. All values lower than the LOD were considered negative reads. Positive detection rates for various mycotoxins at each location are presented in Supplementary Tables S1 and S2 for 2017 and 2018, respectively.

Mycotoxin data were analyzed using PROC GLIMMIX in SAS software (SAS Institute Inc., version 9.4, Cary, NC, USA) at a *p*-value of 0.05. Data analysis was conducted separately by year due to large environmental differences between 2017 and 2018 growing seasons. Location was considered a fixed effect and replication as a random effect in the model. Locations were considered significantly different at *p* < 0.05, and all pairwise comparisons were made using the lsmeans statement with Tukey’s adjustment. Standard deviation for toxins that were different between locations are presented in Supplementary Tables S3 and S4 for 2017 and 2018, respectively.

## Figures and Tables

**Table 2:** Statistics of mycotoxin concentrations from 45 maize grain samples in 2017 across nine locations in Michigan. Percentage of positive samples, the limit of detection (LOD), the limit of quantification (LOQ), mean, standard deviation (SD), minimum, and maximum concentrations were calculated for each mycotoxin.

Mycotoxin	LOD ^a^	LOQ ^b^	Positive ^c^	Mean	Median	SD	Min	Max
	μg kg^−1^	μg kg^−1^	%	–––––––––––––––– μg kg^−1^ ––––––––––––––––				
Beauvericin	0.04	0.10	78	0.11	0.69	0.03	ND ^d^	3217.50
Deoxynivalenol	14.34	30.13	93	1228.65	471.99	1657.95	ND	8288.60
Deoxynivalenol 3-β-D-glucoside	1.44	3.30	73	1195.70	384.79	1631.45	ND	6266.49
15-acetyl-deoxynivalenol	23.24	39.88	47	137.23	0	193.50	ND	927.64
3-acetyl-deoxynivalenol	13.86	31.76	0	-	-	-	-	-
Diacetoxyscirpenol	0.29	0.72	0	-	-	-	-	-
Enniatin A	0.01	0.02	100	0.11	0.11	0.03	0.05	0.19
Enniatin A1	0.01	0.02	0	-	-	-	-	-
Enniatin B	<0.00	0.01	84	0.28	0.30	0.14	ND	0.68
Enniatin B1	<0.00	0.01	4	0.02	0	0.11	ND	0.65
Fumonisin B1	0.19	0.49	80	299.37	10.38	737.82	ND	3686.44
Fumonisin B2	0.97	2.47	80	984.53	21.65	2310.07	ND	22,538.63
Fumonisin B3	0.58	1.55	71	642.24	19.15	1737.81	ND	8733.03
Fusarenon-X	104.19	232.43	0	-	-	-	-	-
HT-2	16.54	30.12	0	-	-	-	-	-
Moniliformin	0.12	0.26	47	45.50	0	104.90	ND	420.45
Nivalenol	-	-	0	-	-	-	-	-
T-2 toxin	0.30	0.69	11	3.42	0	11.70	ND	64.00
Zearalenone	0.03	0.07	69	196.60	9.8	451.59	ND	2204.13

^a^ Limit of detection. Calculated as three times the standard deviation around the analyte retention time ^b^ Limit of quantification. Calculated as ten times the standard deviation around the analyte retention time ^c^ Samples below the limit of detection were considered negative reads ^d^ ND represents samples below the limit of detection.

**Table 3:** Statistics of mycotoxin concentrations from 45 maize grain samples in 2018 across nine locations in Michigan. Percentage of positive samples, the limit of detection (LOD), the limit of quantification (LOQ), mean, standard deviation (SD), minimum, and maximum concentrations were calculated for each mycotoxin.

Mycotoxin	LOD ^a^	LOQ ^b^	Positive ^c^	Mean	Median	SD	Min	Max
	μg kg^−1^	μg kg^−1^	%	–––––––––––––––– μg kg^−1^ –––––––––––––––––				
Beauvericin	0.02	0.07	100	588.58	58.09	1442.40	1.04	7446.21
Deoxynivalenol	58.49	139.66	100	5143.06	4004.76	4910.49	173.82	20,475.00
Deoxynivalenol 3-β-D-glucoside	0.73	1.77	100	757.88	392.58	845.09	7.44	3249.36
15-acetyl-deoxynivalenol	11.64	22.77	100	451.20	276.91	1787.60	38.93	1787.60
3-acetyl-deoxynivalenol	2.51	6.06	64	11.63	9.66	13.36	ND ^d^	63.04
Diacetoxyscirpenol	-	-	0	-	-	-	-	-
Enniatin A	0.01	0.03	11	0.52	0	3.25	ND	21.84
Enniatin A1	0.05	0.10	9	0.65	0	4.06	ND	27.28
Enniatin B	0.01	0.01	20	0.11	0	0.45	ND	2.34
Enniatin B1	0.01	0.02	9	0.21	0	1.19	ND	7.94
Fumonisin B1	0.21	0.50	96	2179.62	324.04	6926.47	ND	45,145.82
Fumonisin B2	0.09	0.23	89	730.75	43.99	2861.31	ND	19,118.06
Fumonisin B3	0.09	0.25	89	700.72	44.89	2693.38	ND	17,972.72
Fusarenon-X	-	-	0	-	-	-	-	-
HT-2 toxin	1.65	0.80	9	14.07	0	52.63	ND	276.74
Moniliformin	0.06	0.13	73	141.25	14.22	267.99	ND	1160.35
Nivalenol	-	-	0	-	-	-	-	-
T-2 toxin	0.30	0.69	27	7.18	0	26.47	ND	156.65
Zearalenone	0.03	0.05	100	592.84	109.26	984.60	0.56	4148.75

^a^ Limit of detection. Calculated as three times the standard deviation around the analyte retention time ^b^ Limit of quantification. Calculated as ten times the standard deviation around the analyte retention time ^c^ Samples below the limit of detection were considered negative reads ^d^ ND represents samples below the limit of detection.

**Table 4:** Effect of location on deoxynivalenol (DON), deoxynivalenol 3-β-D-glucoside (D3G), zearalenone (ZEN), and enniatin A (ENNA) contamination levels in 2017; all other toxins tested did not vary by location.

Location	Mycotoxin			
	DON	D3G	ZEN	ENNA
	––––––––––––––––––––– μg kg^−1^ –––––––––––––––––––––––			
Allegan	1900 ab	2400 ab	386 ab	0.08 b
Branch *	1780 ab	1570 ab	191 ab	0.10 ab
Cass *	1950 ab	2220 ab	143 ab	0.09 b
Huron	130 b	106 b	0.49 b	0.10 ab
Ingham	205 b	171 b	0.36 b	0.14 a
Mason *	485 b	336 b	112 b	0.12 b
Montcalm	1320 ab	769 b	63.9 ab	0.12 ab
Saginaw	126 b	51.5 b	3.39 b	0.14 a
Washtenaw	3690 a	3110 a	869 a	0.14 a
*p*-value	0.002	0.002	0.03	0.0006

Note: Letters indicate significance within columns. Means followed by different letters indicate significant differences at *p* < 0.05. All other mycotoxins did not differ between locations. * indicates locations under irrigation.

**Table 5:** Effect of location on deoxynivalenol (DON), deoxynivalenol 3-β-D-glucoside (D3G), HT-2 toxin (HT-2), and T-2 toxin (T-2) contamination levels in 2018; all other toxins tested did not vary by location.

Location	Mycotoxin			
	DON	D3G	HT-2	T-2
	––––––––––––––––––––– μg kg^−1^ –––––––––––––––––––––			
Allegan	1820 bcd	361 ab	0 b	0.17 b
Branch *	969 d	130 b	0 b	0.62 b
Cass *	3040 bcd	252 ab	0 b	0.17 b
Huron	10,800 a	1150 ab	117 a	55.5 a
Ingham	3870 abcd	741 ab	0 b	0.22 b
Mason *	5870 abcd	148 ab	10.0 b	7.27 b
Montcalm	9190 abc	1000 ab	0 b	0.31 b
Saginaw	9480 ab	1600 a	0 b	0.00 b
Washtenaw	1250 d	103 b	0 b	0.29 b
*p*-value	0.0001	0.006	0.001	0.005

Note: Letters indicate significance within columns. Means followed by different letters indicate significant differences at *p* < 0.05. All other mycotoxins did not differ between locations. * indicates locations under irrigation.

**Table 6:** Total monthly precipitation (mm) and average monthly temperatures (°C) at each of nine locations in Michigan in 2017 and 2018 along with 30-year averages for each location.

Location	2017				2018				30-Year Average			
	July	August	September	Total	July	August	September	Total	July	August	September	Total
	––––––––––––––––––––––––––––––––– Precipitation (mm) –––––––––––––––––––––––––––––––––											
Allegan	57.1	71.6	19.3	148	84.0	129	57.4	270	86.1	97.5	96.8	280
Branch *	82.5	46.0	25.9	154	50.0	113	51.0	214	89.2	108	105	302
Cass *	85.3	68.1	46.4	200	29.7	131	36.3	197	111	103	95.0	309
Huron	65.7	68.0	27.9	162	27.4	133	40.6	201	81.3	89.7	98.6	270
Ingham	46.5	41.9	22.1	111	40.9	87.1	93.7	222	82.8	83.8	92.2	259
Mason *	83.0	45.7	34.5	163	31.5	140	101	273	77.0	90.2	98.6	266
Montcalm	23.3	34.5	17.8	75.6	30.2	196	99.3	326	79.0	92.5	96.3	268
Saginaw	78.7	79.5	21.8	180	29.0	179	62.7	271	79.8	81.0	95.5	256
Washtenaw	38.8	60.4	32.5	132	28.9	83.5	45.4	158	88.4	77.0	87.9	253
	–––––––––––––––––––––––––––––––– Temperature (°C) ––––––––––––––––––––––––––––––––											
Allegan	21.8	19.4	17.7		22.3	22.1	18.5		21.2	20.3	16.1	
Branch *	19.9	17.3	15.8		22.4	22.1	18.8		21.7	20.7	16.4	
Cass *	21.8	19.7	18.4		22.4	22.2	19.0		22.3	21.3	17.1	
Huron	20.8	19.1	17.4		22.1	21.2	17.7		20.7	19.8	15.8	
Ingham	21.6	19.2	17.5		22.2	21.5	17.7		22.1	21.3	16.9	
Mason *	19.6	18.1	17.2		21.4	21.1	17.1		20.7	19.9	15.9	
Montcalm	20.9	18.4	17.0		21.1	21.1	17.1		21.1	20.2	15.9	
Saginaw	22.1	20.1	18.2		23.2	22.6	18.5		21.6	20.69	16.1	
Washtenaw	22.5	20.1	17.9		22.9	22.4	19.0		22.2	21.3	16.7	

Note: Monthly temperature and rainfall numbers obtained from the nearest MSU Enviro-weather station (https://mawn.geo.msu.edu/ (accessed on 1 March 2021). Thirty-year average temperature and precipitation data collected from the National Oceanic and Atmosphere Administration (https://www.ncdc.noaa.gov/cdo-web/datatools/normals (accessed on 1 March 2021). * indicates locations under irrigation.

**Table 7:** Results of ANOVA for western bean cutworm incidence at each of nine locations across Michigan in 2017 and 2018.

Location	2017	2018
	––––––% ––––––––	
Allegan	4.00 b	12.0 a
Branch *	14.0 b	4.00 a
Cass *	29.6 b	3.00 a
Huron	21.1 b	15.0 a
Ingham	19.0 b	8.00 a
Mason *	18.0 b	27.9 a
Montcalm	11.9 b	13.0 a
Saginaw	21.0 b	15.0 a
Washtenaw	95.0 a	27.0 a
*p*-value	<0.0001	0.1195

Note: Letters indicate significance within columns. Means followed by different letters indicate significant differences at *p* < 0.05. * indicates locations under irrigation.

## Data Availability

Not applicable.

## Supplementary Materials

The following supporting information can be downloaded at: https://www.mdpi.com/article/10.3390/toxins14070431/s1. Table S1. Positive detection rates for beauvericin (BEA), deoxynivalenol (DON), deoxynivalenol 3-β-D-glucoside (D3G), 15-acetyl-deoxynivalenol (15-ADON), enniatin A (ENNA), enniatin B (ENNB), enniatin B1 (ENNB1), fumonisin B1 (FB1), fumonisin B2 (FB2), fumonisin B3 (FB3), Moniliformin (MON), T-2, and Zearalenone (ZEN) at all locations in 2017; Table S2: Positive detection rates for beauvericin (BEA), deoxynivalenol (DON), deoxynivalenol 3-β-D-glucoside (D3G), 15-acetyl-deoxynivalenol (15-ADON), 3-acetyl-deoxynivalenol (3-ADON), enniatin A (ENNA), enniatin A1 (ENNA1), enniatin B (ENNB), enniatin B1 (ENNB1), fumonisin B1 (FB1), fumonisin B2 (FB2), fumonisin B3 (FB3), Moniliformin (MON), HT-2, T-2, and Zearalenone (ZEN) at all locations in 2018; Table S3: Standard deviation of deoxynivalenol (DON), deoxynivalenol 3-β-D-glucoside (D3G), HT-2 toxin (HT-2), and T-2 toxin (T-2) contamination levels at all locations in 2017; all other toxins tested did not vary by location. * indicates locations under irrigation; Table S4: Standard deviation of deoxynivalenol (DON), deoxynivalenol 3-β-D-glucoside (D3G), HT-2 toxin (HT-2), and T-2 toxin (T-2) contamination levels at all locations in 2018; all other toxins tested did not vary by location. * indicates locations under irrigation.
