# Supplementary material for: Mycotoxin Co-Occurrence in Michigan Harvested Maize Grain
Source: Toxins (Basel). 2022 Jun 24;14(7):431. doi: 10.3390/toxins14070431 (PMC9324039; doi:10.3390/toxins14070431)
Supplement: Supplementary file 1 [file toxins-14-00431-s001.zip › toxins-1707907-SM.pdf]

toxins

MDPI

Article

Mycotoxin Co-Occurrence in Michigan Harvested Maize Grain

Katlin Fusilier, Martin I. Chilvers, Victor Limay-Rios and Maninder P. Singh

Table S1. Positive detection rates for beauvericin (BEA), deoxynivalenol (DON), deoxynivalenol 3-β-D-glucoside (D3G), 15-acetyl-deoxynivalenol (15-ADON), enniatin A (ENNA), enniatin B (ENNB), enniatin B1 (ENNB1), fumonisin B1 (FB1), fumonisin B2 (FB2), fumonisin B3 (FB3), Moniliformin (MON), T-2, and Zearalenone (ZEN) at all locations in 2017.

**Location**	**DON**	**D3G**	**15-ADON**	**ZEN**	**FB1**	**FB2**	**FB3**	**MON**	**ENNB**	**ENNB1**	**ENNA**	**BEA**	**T2**
	% Positive												
Allegan	100	100	60	80	100	100	100	40	80	0	100	80	0
Branch	100	100	80	100	100	100	100	80	80	0	100	80	0
Cass	100	100	80	100	80	80	80	40	100	0	100	100	0
Huron	60	40	0	40	100	60	80	60	100	20	100	60	0
Ingham	100	100	80	100	100	100	100	80	60	0	100	100	0
Mason	80	60	20	40	60	60	80	60	100	0	100	100	40
Montcalm	100	100	80	80	60	60	60	0	100	20	100	60	40
Saginaw	100	20	0	40	60	60	60	40	80	0	100	60	20
Washtenaw	100	100	100	100	100	100	40	60	80	0	100	100	0

Table S2. Positive detection rates for beauvericin (BEA), deoxynivalenol (DON), deoxynivalenol 3-β-D-glucoside (D3G), 15-acetyl-deoxynivalenol (15-ADON), 3-acetyl-deoxynivalenol (3-ADON), enniatin A (ENNA), enniatin A1 (ENNA1), enniatin B (ENNB), enniatin B1 (ENNB1), fumonisin B1 (FB1), fumonisin B2 (FB2), fumonisin B3 (FB3), Moniliformin (MON), HT-2, T-2, and Zearalenone (ZEN) at all locations in 2018.

**Location**	**DON**	**D3G**	**15-ADON**	**3-ADON**	**ZEN**	**FB1**	**FB2**	**FB3**	**MON**	**ENNA**	**ENNA1**	**ENNB**	**ENNB1**	**BEA**	**HT2**	**T2**
	% Positive															
Allegan	100	100	100	0	100	100	60	60	60	0	0	0	0	100	0	20
Branch	100	100	100	0	100	100	100	100	60	0	20	60	20	100	0	20
Cass	100	100	100	80	100	100	100	100	100	0	0	40	20	100	0	20
Huron	100	100	100	100	100	100	100	100	100	20	0	0	0	100	60	60
Ingham	100	100	100	0	100	100	80	80	80	0	20	60	20	100	0	20
Mason	100	100	100	100	100	60	40	40	0	0	0	20	0	100	20	60
Montcalm	100	100	100	100	100	100	100	100	100	60	60	40	40	100	0	20
Saginaw	100	100	100	100	100	100	100	100	100	0	0	0	0	100	0	0
Washtenaw	100	100	100	0	100	100	100	100	40	0	0	20	0	100	0	20

Table S3. Standard deviation of deoxynivalenol (DON), deoxynivalenol 3-β-D-glucoside (D3G), HT-2 toxin (HT-2) and T-2 toxin (T-2) contamination levels at all locations in 2017, all other toxins tested did not vary by location. * indicates locations under irrigation.

Location	Mycotoxin			
	DON	D3G	ZEN	ENNA
	------------------------------------μg kg^{-1}---			
Allegan	1690	2340	661	0.016
Branch*	1160	13106	197	0.027
Cass*	1600	2200	192	0.010
Huron	197	168	0.668	0.007
Ingham	264	324	0.690	0.031
Mason*	817	616	247	0.034
Montcalm	1070	7262	85.2	0.026
Saginaw	90.0	115	4.73	0.028
Washtenaw	2720	1660	935	0.014

Table S4. Standard deviation of deoxynivalenol (DON), deoxynivalenol 3-β-D-glucoside (D3G), HT-2 toxin (HT-2) and T-2 toxin (T-2) contamination levels at all locations in 2018, all other toxins tested did not vary by location. * indicates locations under irrigation.

Location	Mycotoxin			
	DON	D3G	HT-2	T-2
	-------------------------------------μg kg^{-1}--------------------------------------			
Allegan	1330	418	0.169	0.379
Branch*	1110	142	0.619	1.38
Cass*	1780	117	0.166	0.371
Huron	5720	741	55.5	64.5
Ingham	2990	1200	0.221	0.495
Mason*	1850	1100	7.27	14.1
Montcalm	6170	659	0.314	0.702
Saginaw	5150	793	0	0
Washtenaw	1650	163	0.289	0.647
